# Health-related quality of life of early-stage breast cancer patients after different radiotherapy regimens

**DOI:** 10.1007/s10549-021-06314-4

**Published:** 2021-07-03

**Authors:** Daphne H. M. Jacobs, Ramona K. Charaghvandi, Nanda Horeweg, John H. Maduro, Gabrielle Speijer, Ellen M. A. Roeloffzen, Mirjam Mast, Enja Bantema-Joppe, Anna L. Petoukhova, Desirée H. J. G. van den Bongard, Peter Koper, Anne P. G. Crijns, Corrie A. M. Marijnen, Helena M. Verkooijen

**Affiliations:** 1 Department of Radiation Oncology, Leiden University Medical Center, Albinusdreef 2, PO box 9600, 2300 RC Leiden, The Netherlands; 2 Department of Radiation Oncology, Haaglanden Medical Center, Burgemeester Banninglaan, 2262 BA Leidschendam, The Netherlands; 3 Department of Radiation Oncology, University Medical Center Utrecht, Utrecht, The Netherlands; 4 Department of Radiation Oncology, University Medical Center, Groningen, Groningen, The Netherlands; 5 Department of Radiation Oncology, Radboud University Medical Center, Nijmegen, The Netherlands; 6 Department of Radiation Oncology, Haga Hospital, The Hague, The Netherlands; 7 Department of Radiation Oncology, Isala Clinics, Zwolle, The Netherlands; 8 Department of Radiation Oncology, Radiotherapy Institute Friesland, Friesland, The Netherlands; 9 Department of Radiation Oncology, Amsterdam University Medical Centers, Amsterdam, The Netherlands; 10 Department of Radiation Oncology, Netherlands Cancer Institute, Amsterdam, The Netherlands

**Keywords:** Early-stage breast cancer, Health-related quality of life, Accelerated Partial Breast Irradiation, Intraoperative radiotherapy

## Abstract

**Purpose:**

To evaluate and compare health-related quality of life (HRQL) of women with early-stage breast cancer (BC) treated with different radiotherapy (RT) regimens.

**Methods:**

Data were collected from five prospective cohorts of BC patients treated with breast-conserving surgery and different RT regimens: intraoperative RT (IORT, 1 × 23.3 Gy; *n* = 267), external beam accelerated partial breast irradiation (EB-APBI, 10 × 3.85 Gy; *n* = 206), hypofractionated whole breast irradiation(hypo-WBI, 16 × 2.67 Gy; *n* = 375), hypo-WBI + boost(hypo-WBI-B, 21–26 × 2.67 Gy; *n* = 189), and simultaneous WBI + boost(WBI-B, 28 × 2.3 Gy; *n* = 475).

Women ≥ 60 years with invasive/in situ carcinoma ≤ 30 mm, cN0 and pN0-1a were included. Validated EORTC QLQ-C30/BR23 questionnaires were used to asses HRQL. Multivariable linear regression models adjusted for confounding (age, comorbidity, pT, locoregional treatment, systemic therapy) were used to compare the impact of the RT regimens on HRQL at 12 and 24 months. Differences in HRQL over time (3–24 months) were evaluated using linear mixed models.

**Results:**

There were no significant differences in HRQL at 12 months between groups except for breast symptoms which were better after IORT and EB-APBI compared to hypo-WBI at 12 months (*p* < 0.001). Over time, breast symptoms, fatigue, global health status and role functioning were significantly better after IORT and EB-APBI than hypo-WBI. At 24 months, HRQL was comparable in all groups.

**Conclusion:**

In women with early-stage breast cancer, the radiotherapy regimen did not substantially influence long-term HRQL with the exception of breast symptoms. Breast symptoms are more common after WBI than after IORT or EB-APBI and improve slowly until no significant difference remains at 2 years posttreatment.

**Supplementary Information:**

The online version contains supplementary material available at 10.1007/s10549-021-06314-4.

## Introduction

Since the introduction of breast-conserving treatment as standard of care for patients with early-stage breast cancer, various radiotherapy dose regimens have been used. Initially, whole breast irradiation(WBI) consisted of a dose of 50–60 Gy given over 5–6 weeks with an additional “boost” dose of 16–20 Gy to improve local control [1, 2]. Studies on hypofractionation were conducted, ultimately resulting in a regimen of 40 Gy in 15 daily fractions [3, 4]. More recent studies showed that further hypofractionation of WBI to 26 Gy in five fractions is safe and feasible [5]. Furthermore, due to a lower absolute risk of recurrence as a result of contemporary (neo) adjuvant systemic therapy, a boost is only applied when the local recurrence risk outweighs the increased risk of fibrosis and adverse cosmesis [6].

As oncological outcomes and overall survival for early-stage breast cancer are good and continue to improve, the impact of radiotherapy on quality of life is important. Adverse effects of oncological treatments include but are not limited to fatigue, breast pain, and impaired physical or emotional functioning [7–9].

One strategy to further minimize adverse effects and improve health-related quality of life (HRQL) is to reduce the radiation volume from the whole breast to solely the tumour bed, so called partial breast irradiation. By reducing the irradiated volume, further hypofractionation can be applied thereby also shortening the treatment time, resulting in Accelerated Partial Breast Irradiation ((A)PBI). Several studies have been performed with a two groups comparison of (A)PBI to WBI, showing favourable HRQL in patients treated with (A)PBI [10–13]. Thus, a reduction in overall treatment burden can have a beneficial impact in HRQL of early-stage breast cancer patients. Given various APBI and WBI schedules are available for treatment it is of interest to evaluate how these various radiation techniques impact HRQL. In this study HRQL was compared between large cohorts of patients who were treated with different radiotherapy regimens: WBI with a boost, hypofractionated WBI with or without a boost, external beam APBI and single-fraction intraoperative APBI. We aimed to provide an overview of the impact of different radiation techniques on HRQL of early-stage breast cancer patients.

## Materials and methods

### Patients

Early-stage breast cancer patients were included using the following eligibility criteria: ≥ 60 years, pT1-2 tumours of ≤ 30 mm, cN0 and ≤ pN1a status. Exclusion criteria were: direct axillary dissection (instead of sentinel node procedure), neoadjuvant chemotherapy, or a malignancy (with the exception of non-melanoma skin cancer) 5 years prior to current breast cancer diagnosis.

Eligible patients were selected from the following Dutch prospective cohorts. From 2011 to 2016, patients were treated with intraoperative electron radiotherapy to the tumour bed(IORT cohort, 6–12 MeV, 1 fraction of 23.3 Gy to the surgical bed during surgery; *n* = 267) at the Haaglanden Medical Centre in a cohort study(10–042 METC ZuidwestHolland; NTR2931) [14, 15]. In another study cohort patients received photon external beam accelerated partial breast irradiation (EB-APBI cohort, ten daily fractions of 3.87 Gy; *n* = 206) at the Haga Hospital and Isala clinics. In both these prospective cohorts quality of life questionnaires collected until the 1st of May 2019 were included.

The Utrecht prospective cohort for Multiple BREast cancer intervention studies and Long-term evaluation (UMBRELLA) includes consecutive patients referred for radiotherapy to the Radiation Oncology department of the University Medical Centre Utrecht. Since October 2013 on, clinical data and patient-reported outcomes of breast cancer patients treated with the standard of care have been prospectively collected at predefined timepoints prospectively [16]. For the purpose of this study, we selected patients treated with hypofractionated whole breast irradiation without boost (hypo-WBI cohort; 15 fractions of 2.67 Gy; *n* = 375) or with a boost to the surgical bed (hypo-WBI-B cohort; 21 fractions of 2.67; *n* = 189). Questionnaires collected until January 2019 were included.

The University Medical Centre Groningen prospectively collected data of stage I–III breast cancer patients treated from 2005 to 2012 with three-dimensional conformal radiotherapy with a simultaneous integrated hypofractionated boost as part of breast-conserving therapy (WBI-B cohort, 28 fractions of 1.8 Gy to the whole breast and 2.3–2.4 Gy (depending on surgical margins) boost to the surgical bed, the standard treatment in this period of time, *n* = 475). Questionnaires until 2 years after treatment were included [17].

To compare the HRQL of breast cancer patients in above-mentioned cohorts to those of the general age-matched population, we used the Dutch Profiles data for women aged 60 or older, which represents the HRQL of women aged 60 years or older of a representative sample of the Dutch population in 2013 [18].

### Health-related quality of life

Patients in all cohorts completed the internationally validated European Organization for Research and Treatment of Cancer (EORTC) Quality of Life (QLQ-C30) (version 3) and breast cancer module (BR23) at several timepoints after radiotherapy. Calculation of functioning and symptom scale scores was performed according to the EORTC scoring manual, in which scores are calculated if at least half of questions are available [19]. Relevant functioning and symptom scales were defined before analysis. For functioning scales [Global health score (GHS); physical functioning (PF), role functioning (RF), social functioning (SF), emotional functioning (EF), body image (BRBI)], a higher score (range 0–100) represents better functioning. For symptom scales [fatigue (FA), breast symptoms BRBS)] a higher score represents a higher symptom burden.

### Timepoints

Timepoints defined for this study were: 3, 6, 12 and 24 months after the date of the last radiotherapy fraction. In the IORT cohort this is the day of lumpectomy, as these patients received only one intraoperative fraction. In the other cohorts, the patients underwent their last radiotherapy fraction several weeks after lumpectomy (median EB-APBI: 5 weeks, hypo-WBI: 8, hypo-WBI-B: 9, WBI-B: 11). Questionnaires prior to radiotherapy (i.e. baseline) were not available for the WBI-B cohort. In the other cohorts there was a substantial variety in timing of these questionnaires such that these could not be included in the analyses.

Inclusion of questionnaires for analysis was limited to those being filled out within a reasonable range from the specified timepoints. Hence, between 1.5 and 4.5 months for the 3-month timepoint, between 45 and 9 months for the 6-month timepoint, between 9 and 18 months for the 12-month timepoint and between 18 and 30 months for the 24-month timepoint. In the case of multiple questionnaires available within 1 interval, the one closest to the actual timepoint was used.

### Baseline characteristics and comorbidity

Data on baseline patient, tumour and treatment characteristics were collected prospectively in all cohorts, with exception of comorbidity in three out of five cohorts. Data on comorbidity were collected in all cohorts using different comorbidity scales. In the IORT and EB-APBI cohorts, the Adult Comorbidity Evaluation(ACE)-27 index was prospectively collected; in the hypo-WBI(-B) cohorts the Charlson Comorbidity Index(CCI) was retrospectively collected, and in the WBI-B cohort the National Cancer Institute comorbidity index(NCI) was retrospectively collected [20, 21]. Even though the different comorbidity scores correspond acceptably when categorized into three levels, the ACE-27 and CCI comorbidity indices were retrospectively reconstructed into the NCI comorbidity index to ensure maximum uniformity between cohorts [22, 23]. Thereafter, the score was dichotomized into none/mild (a score of 0 or 1) and moderate/severe (≥ 2 points).

### Endpoints and statistical analysis

The primary endpoint was difference in HRQL between treatment groups at 12 months after treatment, with difference in HRQL at 24 months as a secondary endpoint. Multiple linear regression analyses of the 12 and 24-month HRQL outcomes were performed. Correction for pre-specified confounders was performed: age (60–69 vs ≥ 70 years), comorbidity(none or mild vs moderate or severe according to NCI), planned systemic therapy(none vs endocrine therapy vs chemotherapy vs combination), pT status(pT1 vs pT2 vs pTis), regional radiotherapy of any axillary level 1 through 4 (yes vs no), and axillary lymph node dissection(yes vs no). The hypo-WBI group was the reference group as this is the current standard of care in the Netherlands.

To correct for multiple testing, a two-sided *p* value of ≤ 0.01 was deemed significant. A clinically relevant difference in mean scores was defined according to Osoba et al.; ≥ 5–10 points is small, ≥ 10–20 points is moderate and ≥ 20 is a large clinically relevant difference [24].

The difference in HRQL over time up to 24 months after treatment was a secondary endpoint. Because no HRQL data was collected at 3 and 6 months in the WBI-B cohort, this analysis only included the IORT, EB-APBI, HYPO-WBI and HYPO-WBI-B cohorts. Scales were analysed with linear mixed models with patients included as random effects, and time, treatment and the interaction between time and treatment as fixed effects. The models were corrected for aforementioned confounders.

The four items of the breast symptoms scale (breast pain, oversensitivity, swelling, and skin problems) were dichotomized in “not at all” and “a little” vs. “quite a bit” and “very much”. Generalized estimating equations were used to evaluate these symptoms over time, using the same methods as in the linear mixed models.

All above-mentioned analyses were performed in SPSS version 25 [IBM SPSS statistics for Windows. Armonk, NY: IBM Corp].

Heatmaps were created to visualize HRQL for each patient separately for all functioning scales and fatigue and breast symptoms at 3, 6, 12 and 24 months for all treatment groups. This visualization also facilitates identification of possible correlations between functioning and symptoms scales. Hierarchical cluster analysis according to Ward’s minimum variance method with Euclidean distances as input was performed to plot heatmaps of HRQL at each timepoint. Patients were clustered within the five different treatment groups, which are presented in separate blocks to visualize the impact of treatment across the whole spectrum of HRQL at the individual level. This analysis was performed using R version 3.6.1 (http://www.r-project.org/), package ComplexHeatmap Version 2.0.0.

## Results

### Patients

In total, 1512 patients were included: 267 patients had undergone IORT, 206 EB-APBI, 375 hypo-WBI, 189 hypo-WBI-B, and 475 WBI-B.

Patient, tumour and treatment characteristics are shown in Table 1. There were differences between cohorts regarding comorbidity, prevalence of in situ carcinoma and axillary lymph node dissection.

**Table 1:** Patient, tumour and treatment characteristics per cohort

		IORT		EB-APBI		Hypo-WBI		Hypo-WBI-B		WBI-B	
		*N* = 267		*N* = 206		*N* = 375		*N* = 189		*N* = 475	
		*N*	%	*N*	%	*N*	%	*N*	%	*N*	%
Age	60–69	162	61	123	60	236	63	148	78	294	62
	≥ 70	105	39	83	40	139	37	41	22	181	38
**Comorbidity**	None/mild	186	70	146	71	268	71	144	76	435	92
**NCI**	≥ Moderate	81	30	60	29	103	28	43	23	40	8
	Unknown	0	0	0	0	4	1	2	1	0	0
**pT**	pT1	224	84	153	74	302	80	109	58	409	86
	pT2	26	10	27	13	40	11	22	11	66	14
	pTis	17	6	26	13	33	9	58	31	0	0
pN	pN0	236	88	177	86	314	84	149	79	414	87
	pN1mi	14	5	4	2	19	5	7	4	26	6
	pN1a	5	2	2	2	5	7	8	4	35	7
	NA	12	5	21	10	17	4	25	13	0	
ER	Positive*	233	93	170	94	325	96	89	72	405	88
	NA/Unknown	17		24		37		65		0	
Her2Neu	Negative*	232	94	165	94	322	96	113	92	414	93
	NA/Unknown	19		30		41		66		31	
Systemic	None	156	58	126	61	244	65	106	56	257	54
therapy	HT	91	34	61	30	106	28	42	22	169	36
	CT	5	2	5	2	4	1	16	9	20	4
	Combination	14	5	12	6	21	6	25	13	29	6
	Unknown	1	0	2	1	0	0	0	0	0	0
**Locoregional radiotherapy**	Yes	6	2	0	0	28	8	15	8	6	1
Axillary dissection**	Yes	1	0	2	1	6	2	3	2	63	13

Characteristics that relevantly differ between groups are marked bold

*NA* Not applicable *NCI* National Comorbidity Index, *ER* oestrogen receptor

*Percentage not including “not applicable/unknown”; ** after initial SN procedure

At 12 months, response rates varied from 75 to 92%. There was a difference in the proportion of returned questionnaires between treatment cohorts (Online Table A). Patients who did not return questionnaires at 12 months had more moderate-severe comorbidity(Online Table B), but there were no significant differences in HRQL scales at 6 months between those who did and did not return questionnaires.

### HRQL at 12 and 24 months after treatment

At 12 months, only breast symptoms differed significantly and to a small but clinically relevant extent: patients treated with IORT or EB-APBI reported significantly less breast symptoms compared to patients treated with hypo-WBI-B, WBI-B and hypo-WBI (the reference cohort) in multivariable linear regression (IORT *p* < 0.001 *B* − 5.3 (99% CI − 9.0–1.6); EB-APBI *p* = 0.002, *B* − 4.8 (− 8.8–0.7)). The *B* value states the mean estimate of difference in HRQL scale compared to reference group in the corrected model. The differences in means between IORT treated patients and all WBI cohorts can be read from Table 2 and were clinically relevant to a small extent (difference of 5–10 points). For EB-APBI there was a clinical relevant difference compared to hypo-WBI-B and WBI-B. At 24 months after treatment, breast symptoms had further decreased in all cohorts, and no significant or clinically relevant differences between any of the groups remained.

**Table 2:** Means of patient-reported outcome measures per timepoint and results of multivariate regression

		Mean scores 12 months after treatment					Mean scores 24 months after treatment				
		Hypo-WBI (Ref)	IORT	EB-APBI	Hypo-WBI-B	WBI-B	Hypo-WBI (Ref)	IORT	EB-APBI	Hypo-WBI-B	WBI-B
GHS	Mean	77.94	80.39	80.99	81.02	82.52	79.8	79.95	79.19	84.94	82.48
Functioning											
PF	Mean	83.55	86.18	86.69	87.89	86.26	83.76	85.12	85.07	87.95	86.13
RF	Mean	83.58	86.24	87.63	86.99	87.53	85.71	85.15	88.33	90.06	87.78
SF	Mean	91.05	92.11	92.97	91.84	94.01	92.43	92.51	93.04	95.03	93.71
EF	Mean	84.38	84.54	87.47	87.01	86.25	84.23	85.08	87.88	90.14	86.18
BI	Mean	93.43	93.82	95.55	94.15	95.23	93.13	94.38	95.37	95.42	94.87
Symptom											
FA	Mean	24.15	19.78	19.95	18.8	18.5	21.23	19.77	20.76	15.06	18.13
BRBS	Mean	14.95	**9.43**	**9.96**	15.78	16.33	11.21	7.50	9.49	12.16	12.2
	*p* value		** < 0.001**	**0.002**							

For functioning scales a higher score is better, for symptom scales a lower score is better. Significant differences are marked bold. *p* values are shown in case of a significant difference

*GHS* Global health scale, *PF* physical functioning, *RF* role functioning, *SF* social functioning, *EF*, Emotional functioning, *BI* body image, *FA* fatigue, *BS* breast symptoms, *Ref* reference category hypo-WBI

All treatment groups showed similar or better HRQL compared to the general Dutch population data at 12 and 24 months (Fig. 2).

**Fig. 1:**
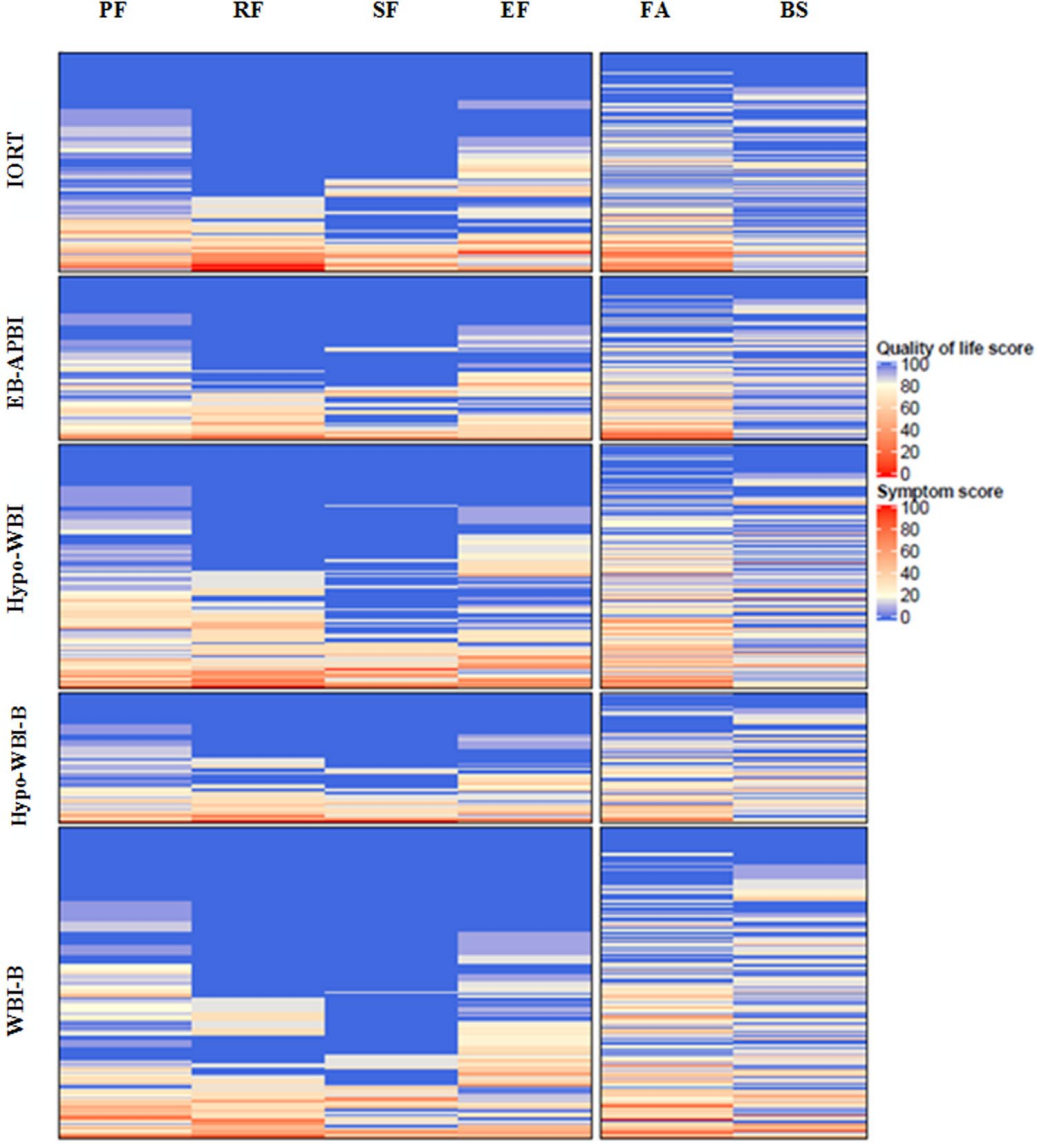
Heatmap of health-related quality of life of individual patients at 12 months after treatment. Physical (PF), Role (RF), Social (SF) and Emotional Functioning (EF), Fatigue (FA) and Breast symptoms (BS) at 12 months per treatment arm. Each horizontal line represents one and the same patient over each scale. A blue colour represents a better outcome, red represents worse outcome. For example, if a horizontal line is blue across all scales, this represents a patient who reports excellent quality of life regarding all of these scales. If a horizontal line is red across all of these scales, this represents a patients who reports poor quality of life regarding all of these scales. If a horizontal line is red across all functioning scales but blue in the breast symptom scale, this represents a patient with poor functioning but no breast symptoms

Quality of life of each individual patient at 12 months is visualized in heatmaps in Fig. 1 and at 3, 6 and 24 months in Online figure A. Each row in the heat map represents an individual patient and the colours indicate the amount of symptoms and level of functioning. In all treatment groups there was a proportion of patients with excellent scores on all HRQL scales and a small proportion with very poor scores in all scales. There is a clear correlation between level of functioning and fatigue. Breast symptoms however, seem uncorrelated and more randomly distributed amongst patients.

### Course of HRQL after treatment

**Fig. 2:**
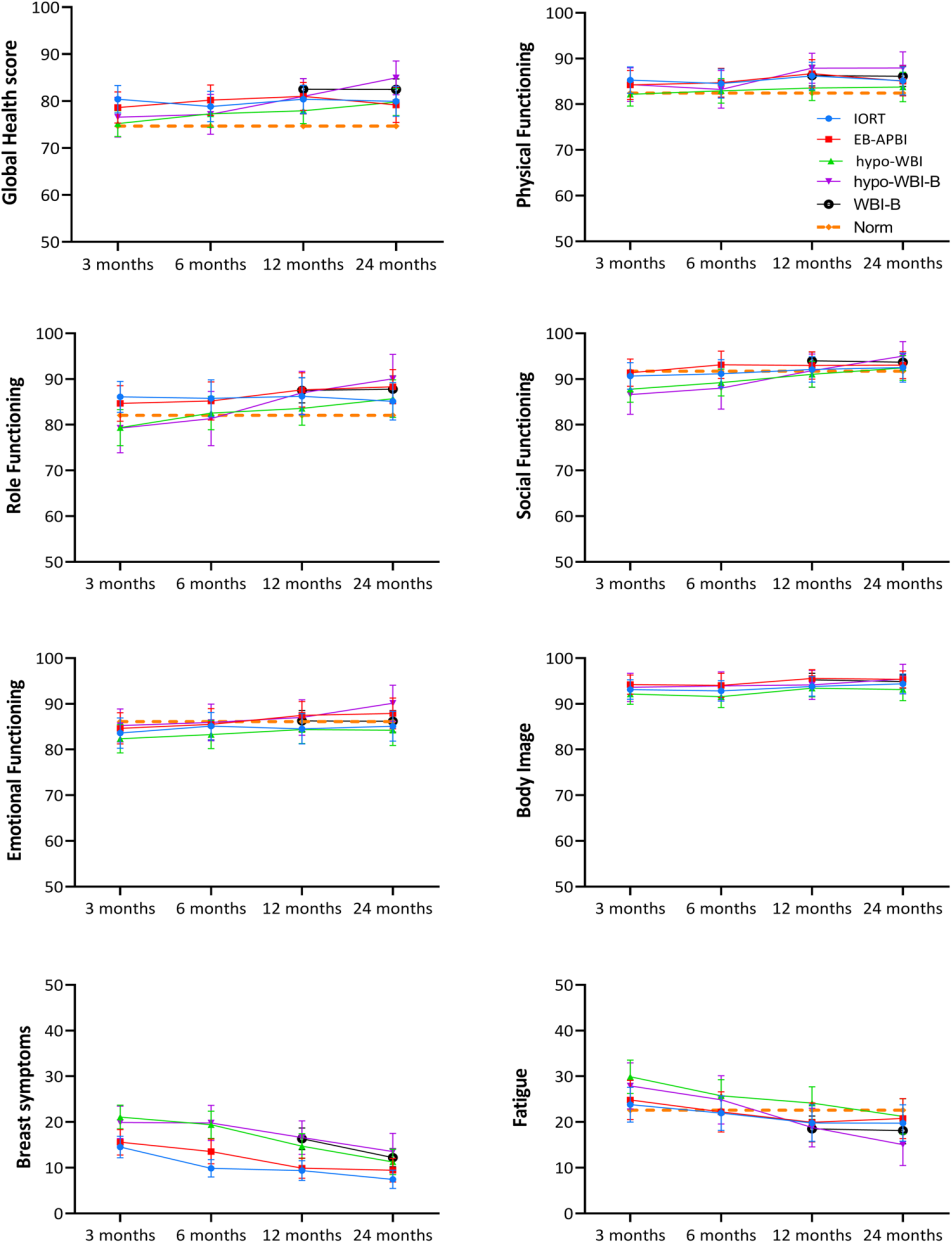
Course of Health-Related Quality of Life over time after four different radiotherapy regimens**.** Mean scores per treatment cohort per timepoint with lines representing 99% confidence intervals are shown. The scores can range from 0 to 100. Note that for functioning scales the vertical axis ranges from 50 to 100 and a higher score represents a better functioning, and for symptom scores the vertical axis ranges from 0 to 50 and a lower score represents a better functioning. The mean of the Dutch age-matched population (‘Norm”) is represented by the orange dotted line

The pattern of HRQL over time between groups in terms of in global health, role functioning, fatigue and breast symptoms was different between the treatment cohorts (Fig. 2; Table 3). Patients treated with IORT or EB-APBI reported better functioning and less symptoms at 3 months compared than those treated with hypo-WBI and hypo-WBI-B.

**Table 3:** Results of linear mixed model analysis illustrating the changes of health-related quality of life up to 24 months after treatment

	GHS	PF	RF	SF	BRBS	FA
	B (99% CI)	B (99% CI)	B (99% CI)	B (99% CI)	B (99% CI)	B(99%CI)
Treatment						
Hypo-WBI	Ref.	Ref.	Ref.	Ref.	Ref.	Ref.
IORT	5.3 (1.5–9.2)***	2.9 (− 0.7–6.6)	6.9 (1.9–11.8)***	2.8 (− 1.0–6.6)	− 6.0 (− 9.4–2.6)***	− 6.3(− 11.1–1.4)**
EB-APBI	4.2 (0.0–8.4)*	2.3 (− 1.6–6.3)	6.0 (0.6–11.4)**	4.1 (− 0.0–8.3)*	− 5.2 (− 8.9–1.5)***	− 5.7(− 11.0–0.5)**
Hypo-WBI-B	2.6 (− 2.1–7.2)	1.8 (− 2.6–6.1)	1.7 (− 4.2–7.7)	0.2 (− 4.3–4.8)	− 1.5 (− 5.6–2.6)	− 3.4(− 9.2–2.5)
Timepoint						
3 M	Ref.	Ref.	Ref.	Ref.	Ref.	Ref.
6 M	2.4 (− 0.4–4.9)	0.4 (− 1.3–2.0)	3.3 (0.10–6.4)**	1.9 (− 0.6–4.3)	− 0.9 (− 3.1–1.2)	− 4.3(− 7.1–1.5)***
12 M	3.3 (0.7–6.0)**	1.2 (− 0.6–3.0)	4.5 (1.0–7.9)**	3.6 (0.9–6.2)**	− 5.7 (− 8.1–3.4)***	− 6.0(− 9.0–3.0)***
24 M	5.4 (2.4–8.3)***	0.7 (− 1.3–2.7)	6.2 (2.3–10.1)***	5.3 (2.2–8.3)***	− 8.4 (− 11.0–5.7)***	− 9.1(− 12.4–5.7)***
T*t						
*p* value	0.001	0.610	0.001	0.034	0.004	0.010

Results of linear mixed model analysis with correction for confounders

*Ref* reference category, *GHS* global health score *PF* physical functioning, *RF* role functioning, *SF* social functioning, *BRBS* breast symptoms, *FA* fatigue, *M* months, T*t interaction between Treatment and time. *B* Mean estimate of difference in HRQL scale compared to reference group in corrected model

For GHS, PF, RF and SF a positive difference is better compared to the reference category, for BRBS and FA a negative difference is better compared to the reference category. Significant differences are marked with asterisks: *p value 0.01 **p value 0.009–0.001 ***p value < 0.001. All significant differences represent significant better scores compared to the reference category

Global health status and role functioning of patients treated with IORT or EB-APBI remained relatively stable over time, whereas for patients treated with WBI an improvement over time was observed. At 24 months, global health status was comparable in all patients. Patients treated with IORT and EB-APBI reported the least fatigue. Fatigue improved over time after all treatments; the largest improvement was seen after hypo-WBI-B. Breast symptoms were less common after IORT and EB-APBI than after WBI techniques, this difference was observed from as early as 3 months after treatment. In all patients breast symptoms decreased from 3 months until 2 years after treatment.

**Fig. 3:**
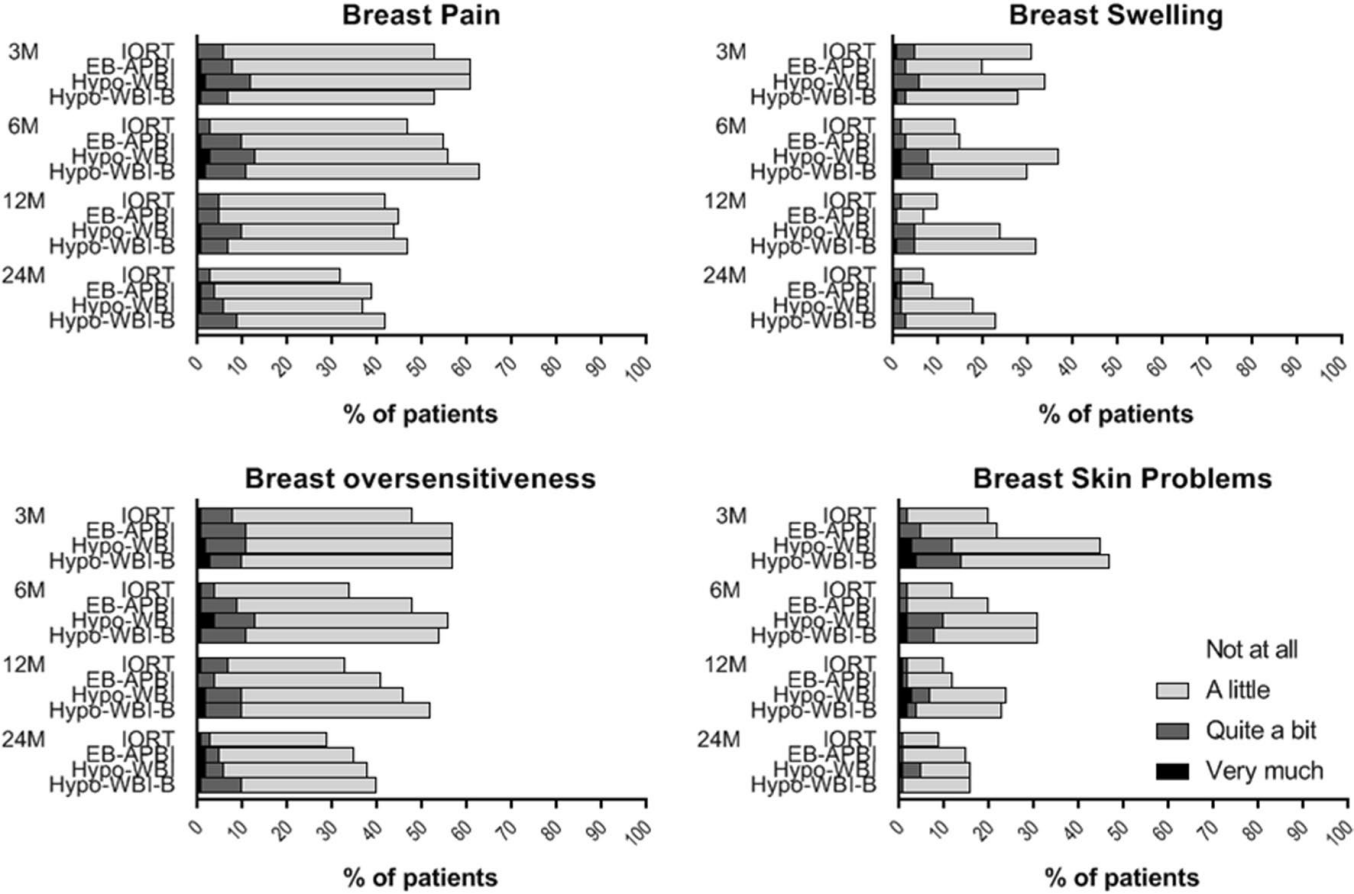
Breast symptoms after four different radiotherapy regimens. Percentage of patients reporting “not at all”, “a little”, “quite a bit” or “very much” symptom burden at different timepoints per treatment group. *M* months after treatment

Regarding the separate symptoms that constitute the breast symptoms scale; only a small fraction of patients experienced a substantial (“quite a bit” or “very much”) symptom burden (Fig. 3). The proportion of patients with symptoms was smallest after IORT and EB-APBI and largest after hypo-WBI-B and hypo-WBI. Over time, skin symptoms were significantly better in patients treated with IORT compared to hypo-WBI (*p* < 0.001; Odds ratio 0.2). There were no other significant differences between treatment groups over time.

Influence of baseline patient and treatment factors on HRQL varied. Older age, at least moderate or severe comorbidity and a combination of endocrine and chemotherapy had a negative influence on most functioning and symptoms scales (Online Table C).

## Discussion

This study, comparing different radiotherapy regimens after breast-conserving surgery in early-stage breast cancer patients of 60 years and older, demonstrates that radiotherapy regimen after breast-conserving surgery has little impact on health-related quality of life. Patients receiving partial breast irradiation experienced significantly less breast symptoms than those treated with whole breast irradiation (with or without boost) at 1 year after treatment. In all treatment groups, breast symptoms improved with no significant difference remaining at 2 years after treatment. Across the first two years after treatment, HRQL was comparable to that of an age-matched general population sample.

Radiotherapy after breast-conserving surgery may impact on HRQL depending on the extensiveness of the regimen in terms of treatment time and volume [25]. A population-based survey study investigating quality of life of elderly (> 67 years) breast cancer patients with treatment regimens ranging from mastectomy to lumpectomy with APBI found that HRQL tended to be better in patients treated with less irradiation (in volume and treatment time) and less surgery at 6 years after treatment [26]. The randomized START trials showed that WBI patients treated with hypofractionated WBI experienced equal or less adverse effects than those treated with conventional WBI [4]. Our study shows as well that less burdensome treatment leads to less impact on HRQL. Patients treated to a limited volume in a few fractions by ABPI had less breast symptoms than patients treated with WBI. They also reported better global health and functioning, but the difference was too small to lead to clinically relevant differences.

Another question is whether the impact on HRQL in general and breast symptoms in particular is attributable to reduction in volume, dose, or both. Results of randomized studies comparing (A)PBI to WBI have in common that patients treated with (A)PBI experience less breast symptoms than patients treated with WBI [10, 12, 27, 28]. An exception is external beam APBI delivered twice daily, which lead to adverse cosmesis and toxicity in a large randomized trial [29]. The IMPORT-LOW study randomized patients to hypofractionated WBI or external beam PBI both in 15 fractions of 2.67 Gy and showed that patients treated with PBI experienced significant less change in appearance of the breast up until 5 years after treatment [10]. This study was the first to confirm that reducing the irradiated volume improves breast appearance, as they did not further accelerate the PBI dose per fraction. Our findings also show less breast symptoms in patients treated to reduced volume of the breast, even though in these cohorts the fraction dose was substantially higher. Remarkably, breast symptoms were less despite the higher fraction dose with APBI techniques (online figure B). As this a retrospective analysis, we cannot definitively attribute the differences to reduced volume and not to longer treatment time. Nonetheless, the finding that there were no relevant differences within the different APBI and WBI groups, even though treatment duration and fraction dose varied substantially suggests that irradiated volume has more impact than treatment duration on long-term breast symptoms.

Shorter treatment duration can also reduce impact on short term HRQL as it might shorten the time it takes a patient to recover from the intensive period of treatment. Several studies have shown quick recovery in patients treated with accelerated compared to conventional radiotherapy [12, 14, 30]. From our data it seems that patients treated with APBI recover faster, within 3 months after treatment, than patients treated with hypo-WBI, possibly explained by a shortened treatment time.

Even though HRQL after oncological treatment is of paramount importance, it remains necessary to weigh the benefits regarding HRQL against oncological outcome. Published studies comparing oncological outcome after (A)PBI and WBI show non-inferiority regarding local recurrence, with higher recurrence rates after (A)PBI reported in a minority of studies [10, 13, 31]. A recent meta-analysis reported a significantly higher local recurrence risk in patients treated with APBI compared to WBI, but no difference in survival [32]. This emphasizes the importance of correct patient selection for (A)PBI.

### Strengths and weaknesses

This study is unique because it assesses the effect of various prospective radiotherapy cohorts on HRQL of breast cancer patients by comparing patients treated with regimens ranging from one fraction IORT to the tumour bed to up to 28 fractions on the whole breast with an integrated boost. This provides unique insight in whether and how de-intensification of radiotherapy influences patients’ HRQL. Furthermore, the fluctuation in HRQL at different timepoints after all treatments emphasizes the need for systematic evaluation of patient-reported outcomes in breast cancer treatment follow up.

Our heatmaps provide a novel view on HRQL of individual patients revealing that patients experiencing burdensome breast symptoms don’t necessarily have a reduced quality of life, and patients experiencing fatigue often have worse levels of functioning.

A weakness is that this study is based on patient data from different prospective cohorts. Any differences in patient and tumour characteristics and treatment time periods between the cohorts may bias the HRQL outcomes. By predefining eligibility criteria and dichotomizing patients’ and tumour characteristics we corrected for differences between cohorts. Nonetheless, several baseline characteristics differed between cohorts, such as comorbidity and the use of chemotherapy, which may influence HRQL outcomes (Table 1; Online Table C). These factors were added to the models to correct for confounding.

Although the investigated cohorts represent relevant radiotherapy treatment modalities over time, we did not have access to cohorts with patients treated with further hypofractionated WBI regimens (i.e. 26 Gy in 5 fractions) as these have only recently been included in routine clinical practice [5]. Also, the moment of the pre-treatment questionnaire collection in the cohorts was too diverse to include this timepoint in our analysis. The timepoint 3 months after treatment was used as the baseline measurement in longitudinal analysis as we were interested in the longer-term effects of treatment in HRQL. Since we utilized predefined eligibility criteria, thereby aiming to level baseline patient and tumour characteristics, and corrected for confounding factors in the analyses we believe our results are robust enough to illustrate changes of HRQL over time across various radiotherapy schedules. Therefore our results are relevant for shared decision making in clinical practice with a variety of radiotherapy schedules available for patients with low-risk breast cancer.

## Conclusion

In conclusion, patients treated with accelerated partial breast irradiation techniques experienced less breast symptoms compared to patients treated with whole breast irradiation techniques up to 1.5 year following treatment. However, the type of radiotherapy regimen did not substantially influence long-term HRQL in patients with early-stage breast cancer above 60 years of age. This information can be useful when counselling patients at low risk of recurrence for radiotherapy, in whom partial breast irradiation is a valuable alternative to whole breast irradiation for low-risk patients.

## Supplementary Information

Below is the link to the electronic supplementary material.

Supplementary file1 (DOCX 4631 kb)

## Data Availability

The datasets generated during and/or analysed during the current study are not publicly available.
