# Supplementary material for: Health-related quality of life of early-stage breast cancer patients after different radiotherapy regimens
Source: Breast Cancer Res Treat. 2021 Jul 3;189(2):387–98. doi: 10.1007/s10549-021-06314-4 (PMC8357747; doi:10.1007/s10549-021-06314-4)
Supplement: Supplementary file 1 — Supplementary file1 (DOCX 4631 kb) [file 10549_2021_6314_MOESM1_ESM.docx]

**Supplementary Material**

**Table A.**

**Appendix table A. Questionnaire response rates per treatment cohort per time point.**

|  | **IORT n=267** | **EB-APBI n=206** | **Hypo-WBI n=375** | **Hypo-WBI-B n=189** | **WBI-B n=475** | **Total  n=1512** |
| --- | --- | --- | --- | --- | --- | --- |
| 3 months | 252/267  (94.4%) | 188/206  (91.3%) | 301/375 (80.3%) | 147/189  (77.8%) | n.a. | 888/1037  (85.6%) |
| 6 months | 253/267  (94.8%) | 187/206  (90.8%) | 284/375  (75.7%) | 135/189  (71.4%) | n.a. | 859/1037  (82.8%) |
| 12 months | 246/267 (92.1%) | 186/206  (90.3%) | 277/370  (74.9%) | 148/187  (79.1%) | 427/475  (89.9%) | 1284/1505  (85.3%) |
| 24 months | 229/267  (85.8%) | 171/206  (83%) | 192/298  (64.4%) | 103/144  (71.5%) | 433/475  (91.2%) | 1128/1390  (81.2%) |

Percentages are extrapolated from the entire cohort. Not all patients in Hypo-WBI and Hypo-WBI-B cohorts had reached 2 year follow up at time of analysis. n.a. = not applicable.

**Table B.**

**Patient, tumour and treatment characteristics of patients with 1 year follow up with (compliant) and without (non-compliant) a returned 1 year questionnaire.**

|  |  |  | |  | |  |
| --- | --- | --- | --- | --- | --- | --- |
|  |  |  | |  | |  |
|  |  | Non-compliant  N=221 % | | Compliant  N=1284 % | | p-value |
| Age | 60-69 | 64% |  | 64% |  | 0.996 |
|  | ≥70 | 36% |  | 36% |  |  |
| Comorbidity | None/mild | 71% |  | 80% |  | 0.003 |
| NCI | ≥Moderate | 29% |  | 20% |  |  |
| pT | pT1 | 79% |  | 79% |  | 0.904 |
|  | pT2 | 13% |  | 12% |  |  |
|  | pTis | 8% |  | 9% |  |  |
| pN | pN0 | 89% |  | 90% |  | 0.757 |
|  | pN1mi | 5% |  | 5% |  |  |
|  | pN1a | 6% |  | 5% |  |  |
| ER | Positive | 92% |  | 90% |  | 0.539 |
| Her2Neu | Negative | 96% |  | 94% |  | 0.259 |
| Systemic | None | 59% |  | 59% |  | 0.993 |
| therapy | HT | 31% |  | 31% |  |  |
|  | CT | 3% |  | 3% |  |  |
|  | Combination | 7% |  | 7% |  |  |
| Locoregional radiotherapy | Yes | 5% |  | 3% |  | 0.258 |
| Axillary dissection | Yes | 5% |  | 5% |  | 0.741 |

| **Table C. Results of linear mixed model analysis illustrating the effect of patient and treatment characteristics on Health-related quality of life up to 24 months after treatment.** | | | | | | | | |
| --- | --- | --- | --- | --- | --- | --- | --- | --- |
|  | |  | **GHS** | **PF** | **RF** | **SF** | **BRBS** | **FA** |
|  |  | | **B(99%CI)** | **B(99%CI)** | **B(99%CI)** | **B(99%CI)** | **B(99%CI)** | **B(99%CI)** |
| **Age** |  | |  |  |  |  |  |  |
| 60-69 |  | | Ref | Ref | Ref | Ref | Ref | Ref |
| >=70 |  | | -2.9(-5.6–-0.3)** | -7.3(-10.1--4.6)*** | -3.6(-7.0--0.2)** | -0.6(-3.2–2.0) | 0.1(-2.2–2.5) | 3.8(0.3–7.3)** |
| **Comorbidity** |  | |  |  |  |  |  |  |
| None/mild |  | | Ref | Ref | Ref | Ref | Ref | Ref |
| Moderate/severe |  | | -7.3(-10.0–-4.5)*** | -9.7(-12.6--6.8)*** | -9.4(-13.0--5.8)*** | -5.9(-8.6--3.2)*** | 2.5(0.1–5.0)* | 10.0(6.3–13.7)*** |
| **Systemic Therapy** | | |  |  |  |  |  |  |
| None |  | | Ref | Ref | Ref | Ref | Ref | Ref |
| HT |  | | -2.0(-5.0–1.0) | -1.4(-4.6–1.7) | -2.6(-6.4–1.3) | -1.3(-4.2–1.7) | -0.5(-3.2–2.2) | 3.3(-0.7–7.3) |
| CT |  | | -10.8(-18.6–-3.1)*** | -5.5(-13.7–2.8) | -16.3(-26.3--6.3)*** | -10.8(-18.3--3.2)*** | 3.4(-3.6–10.3) | 13.5(3.22–23.8)* |
| Combination |  | | -10.4(-15.9--5.0)*** | -9.3(-15.0--3.6)*** | -16.0(-23.0--9.0)*** | -9.2(-14.5--3.9)*** | 1.3(-3.5–6.2) | 15.1(7.9–22.3)*** |
| **Axillary Dissection** | | |  |  |  |  |  |  |
| No |  | | Ref | Ref | Ref | Ref | Ref | Ref |
| Yes |  | | -1.9(-13.3–9.5) | 3.8(-8.1–15.7) | 3.3(-11.4–18.0) | -5.8(-16.9–5.4) | 7.5(-2.6–17.7) | 0.9(-15.8–14.3) |
| **Locoregional RT** | | |  |  |  |  |  |  |
| No |  | | Ref | Ref | Ref | Ref | Ref | Ref |
| Yes |  | | -5.7(-11.9–0.5) | -7.2(-13.8--0.8)** | -4.3(-12.3–3.7) | -4.3(-10.4–1.8) | -0.9(-6.4–4.6) | 8.4 (0.2–16.6)** |
| **pT** |  | |  |  |  |  |  |  |
| pT1 |  | | Ref | Ref | Ref | Ref | Ref | Ref |
| pT2 |  | | 0.8(-3.4–5.0) | -0.9(-5.3–3.5) | -0.3(-5.7–5.1) | -0.1(-4.3–4.0) | -0.6(-4.4–3.2) | 0.9(-4.7–6.5) |
| pTis |  | | -0.8(-4.9–3.2) | -1.3(-5.6–3.0) | -2.6(-7.8–2.7) | -0.6(-4.5–3.4) | 1.6(-2.1–5.2) | 2.2(-3.2–7,5) |

Results of linear mixed model analysis. For effect of radiotherapy treatment and time on HRQL, see table 3 in the manuscript. Ref: reference category; GHS: Global health score; PF: Physical functioning; RF: Role functioning; SF: Social functioning; BRBS: Breast symptoms; FA: Fatigue; M: Months; T*t: interaction between Treatment and time. B: Mean estimate of difference. For GHS, PF, RF and SF a positive difference is better compared to the reference category, for BRBS and FA a negative difference is better compared to the reference category. * p-value 0.01 **p-value 0,009-0,001 ***p-value <0,001.. All significant differences represent significant better scores compared to the reference category.

**Figure A**. **Heatmap of health-related quality of life of individual patients at 3, 6 and 24 months after treatment**

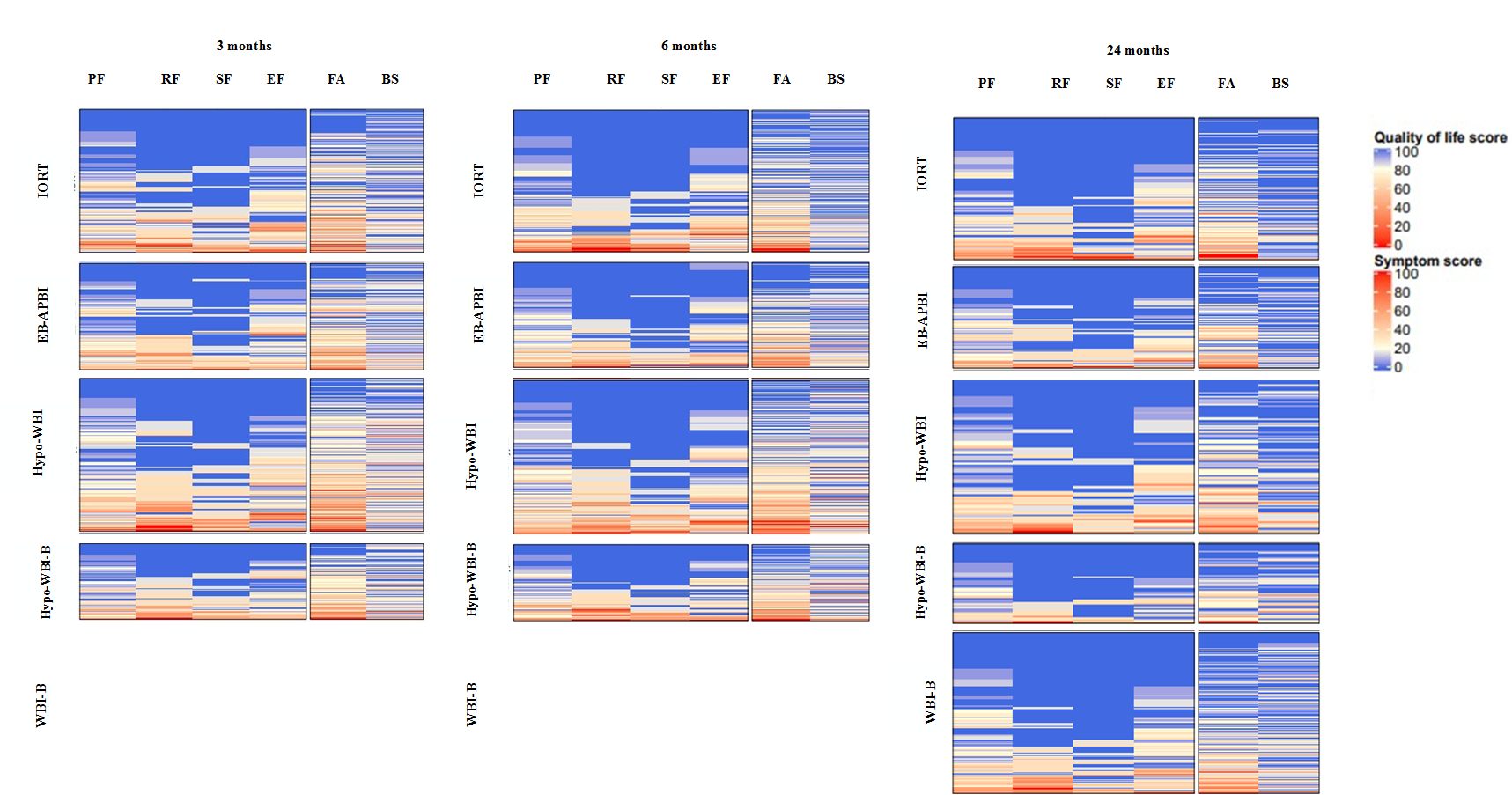


Physical (PF), Role (RF), Social (SF) and Emotional Functioning (EF), Fatigue (FA) and Breast symptoms (BS) at 12 months per treatment arm. Each horizontal line represents one and the same patient over each scale. A blue colour represents a better outcome, red represents worse outcome.

For example, if a horizontal line is blue across all scales, this represents a patient who reports excellent quality of life regarding all of these scales. If a horizontal line is red across all of these scales, this represents a patients who reports poor quality of life regarding all of these scales. If a horizontal line is red across all functioning scales but blue in the breast symptom scale, this represents a patient with poor functioning but no breast symptoms.

**Figure B.** Breast symptoms over time after partial or whole breast irradiation


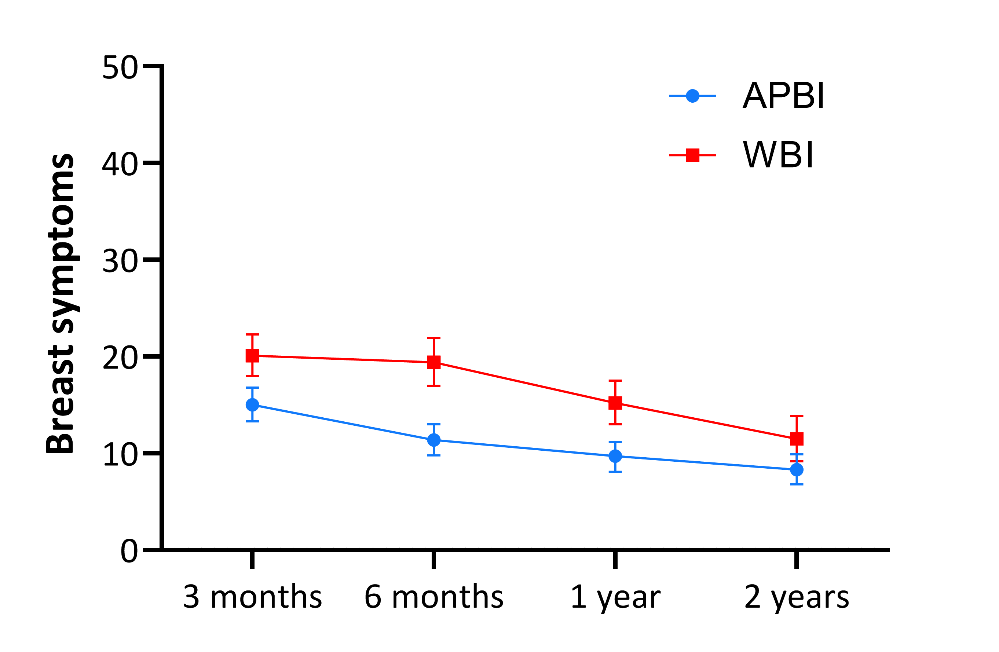


Means of breast symptoms per timepoint for APBI (IORT and EB-APBI) and WBI (hypo-WBI and hypo-WBI-B) with 99% confidence intervals. Lower score represents lesser symptom burden.
